# Dental Nomenclature

**Published:** 1867-01

**Authors:** 


### DENTAL NOMENCLATURE.

HAVING for a long time felt the want of some words in our literature to express Pathological and other conditions of the Dental organs, I have *manufactured* a few, which I think may be used with much convenience. I know that more or less of them, as indeed all of them are open to criticism; and in truth no new words ought to be adopted by the profession without discussion, and I shall therefore expect and wish it. In fact, if the *introduction* of these words to the profession shall have the effect to introduce other and better words which shall be adopted, I shall be more gratified than with the adoption of the following:

Exodontosis.—Dental Exastosis.
Dentalgia.—Toothache, Odontalgia.
Dentinitis.—Inflammation of Dentine.
Dentinalgia.—Pain in the Dentine.
Dentology.— { Discourse about the Teeth. History of the Teeth. }
Dentography.—Description of the Teeth.
Pulpitis.—Inflammation of the Dental Pulp.
Pulpalgia.—Pain in the Dental Pulp.
Peri-cementum.—Alveola-dental Periosteum.
Peri-cementitis.—Inflammation of the above.
Peri-cementalgia.—Pain in the Dental Periosteum.

HENRY S. CHASE.

IOWA CITY, *Nov.*, 1866.

REMARKS:—The above may strike some of our readers as a step in the right direction. As a general rule, however, we have far too many big words. What would be gained by the adoption of most of these words? Take for example "pulpitis." Is inflammation of the pulp different from that of similar tissues in other situations? If not, the new term only confuses. Inflammation of the stomach is a much more satisfactory term than *gastritis*, as is proved by its being far more frequently used. But it may be well enough for the Dental profession to show that we can use as big words as anybody. W.

